# Supplementary material for: Selective depletion of metastatic stem cells as therapy for human colorectal cancer
Source: EMBO Mol Med. 2018 Sep 6;10(10):e8772. doi: 10.15252/emmm.201708772 (PMC6180303; doi:10.15252/emmm.201708772)
Supplement: Supplementary file 1 — Appendix [file EMMM-10-e8772-s001.pdf]

APPENDIX INFORMATION

Selective depletion of metastatic stem cells as therapy for human colorectal cancer

María Virtudes Céspedes, Ugutz Unzueta, Anna Aviñó, Alberto Gallardo, Patricia Álamo, Rita Sala, Alejandro Sánchez-Chardi, Isolda Casanova, María Antònia Mangues, Antonio Lopez-Pousa, Ramon Eritja, Antonio Villaverde, Esther Vázquez and Ramon Mangues.

APPENDIX MATERIAL

Appendix Methods

Appendix Table S1

Appendix Figures S1-10

APPENDIX METHODS

Synthesis of 5'-dimethoxytrityl-5-fluoro-2'-deoxyuridine

5-Fluoro-2'-deoxyuridine (FdU) (3.2 mmol) was reacted with dimethoxytrityl chloride (4.4 mmol) in dry pyridine. The solution was stirred overnight and the solvent was evaporated. The residue was purified by column chromatography using a gradient from pure CH_2Cl_2 to 10% methanol in CH_2Cl_2 to afford the desired dimethoxytrityl-5-fluoro-2'-deoxyuridine (DMT-FdU) in 65%.

Synthesis of 5'-Dimethoxytrityl-5-fluoro-2'-deoxyuridine phosphoramidite

5'-Dimethoxytrityl-5-fluoro-2'-deoxyuridine (1 mmol) was dried by evaporation with *anhydrous* acetonitrile under reduced pressure. Next the product was dissolved in *anhydrous* CH_2Cl_2 (20 mL) under argon and diisopropylethylamine (5 mmol) was added with exclusion of moisture. The solution was cooled with an ice bath and 2-cyanoethoxy-*N*,*N*-diisopropylamino-chlorophosphine (2 mmol) was added dropwise with a syringe. The solution was stirred at room temperature for 1 h and, then, the solution was diluted with CH_2Cl_2 and washed with saturated aqueous NaCl. After drying the organic phase with anhydrous Na_2SO_4, the solvent was evaporated and the product was purified by column chromatography. The column was packed with silica gel using a 10% tritehylamine solution in ethyl acetate/ hexane 1:1 and the compound was eluted with a gradient from ethyl acetate /hexane 1:1 to pure ethyl acetate. The desired phosphoramidite was obtained in 70% yield.

Preparation of the Solid Support functionalized with 5'-dimethoxytrityl-5-fluoro-2'-deoxyuridine

The DMT-FdU derivative (0.4 mmol) was dried by evaporation with *anhydrous* acetonitrile and reacted with succinic anhydride (0.6 mmol) and *N*,*N*-dimethylaminopyridine (0.6 mmol) in CH_2Cl_2 (20 mL). The reaction mixture was stirred overnight at room temperature. The reaction mixture was diluted with 30 mL of CH_2Cl_2 and the solution was washed with saturated *aqueous* NaCl, 10% citric acid *aqueous* solution and again with saturated aqueous NaCl. The organic layer was dried over *anhydrous* Na_2SO_4 and evaporated to dryness. The resulting DMT-FdU hemisuccinate was obtained as a white solid (89% yield) and was used in the next step without further purification.

The DMT-FdU hemisuccinate derivative was incorporated on a long-chain alkylamine-controlled pore glass support (LCAA-CPG) as described in Gupta,1995. Amino-LCAA-CPG (CPG New Jersey; 450 mg, 70 µmol amino/g) was placed into a polypropylene syringe fitted with a polypropylene disc and washed sequentially with methanol, CH_2Cl_2 and acetonitrile. Then 2,2´-dithio*bis*(5-nitropyridine) (0.18 mmol) dissolved in 0.6 mL of a mixture of acetonitrile/ CH_2Cl_2 (1:3) was added to a solution of DMT-FdU hemissuccinate (0.9 mmol) and N.N-dimethylaminopyridine (0.18 mmol) in acetonitrile (1.5 mL). Next, triphenylphosphine (0.18 mmol) was added and the mixture was stirred for a few seconds, added to the support and allowed to react for 1 h. The support was washed with methanol, CH_2Cl_2 and acetonitrile and dried under vacuum. The functionality of the resin was determined by DMT quantification (42 µmol/g). Finally, the solid support was treated with an acetic anhydride solution during 30 min to cap the unreacted amino groups.

Synthesis of the Floxuridine oligonucleotide (oligo-FdU)

Two pentanucleotide sequences were prepared: 1) 5'-$(FdU)_5$-3' is the control FdU pentamer: and 2) 3'-thiolated FdU pentamer: 5'-$(FdU)_5$- HEG-propyl-SH 3' with FdU 5-fluoro-2'-deoxyuridine and HEG as hexaethylenegycol spacer.

To synthetize the control 5'-$(FdU)_5$- 3' pentamer, we used a controlled pore glass (CPG) support functionalized with DMT-FdU prepared as described above. Then, the control pentamer sequence was assembled on a DNA synthesizer (392 Applied Biosystems, Foster City, CA, USA) using a 1 µmol synthesis cycle by successive additions of DMT-protected FdU phosphoramidite. After assembling of the sequence, oligonucleotide support was treated with aqueous ammonia (32%) for 2 hrs at room temperature and the resulting product was purified by HPLC. HPLC conditions: column X-bridgeTM OST C18 (10x50 mm, 2.5 µm); 20 min linear gradient from 0 % to 40%, flow rate 2 mL/min; solution A was 5% acetonitrile in 0.1 M *aqueous* triethylammonium acetate(TEAA) and solution B 70% acetonitrile in 0.1 M *aqueous* TEAA. Pentamer was characterized by mass spectrometry (MALDI-TOF).

Several batches of pentamer FdU oligonucleotide were synthesized in 1 µmol scale on an automated RNA/DNA synthesizer using β-cyanoethylphosphoramidite chemistry and following standard protocols. 3'-Thiol-Modifier C3 solid support (Link Technologies, Lanarkshire, Scotland) was used for the introduction of the thiol group at the 3'-end, then hexaethyleneglycol phosphoramidite (Glen Research) was used as spacer. Finally, the synthesis was completed by repetitive additions of the DMT-protected FdU phosphoramidite. After the assembly of the sequence, oligonucleotide support was treated with aqueous ammonia (32%) with 0.1 M 1,4-dithiothreitol (DTT) for 2h at room temperature. The ammonia solution was concentrated to dryness and the product was desalted on NAP-10 (Sephadex G-25) columns eluted with water prior to use. The purity of the pentamer FdU-HEG-SH was analyzed by HPLC using the conditions described above (see appendix Fig. S1). Pentamer was quantified by absorption at 260 nm and confirmed by MALDI mass spectrometry (MALDI-TOF).

Characterization of the T22-GFP-H6-FdU therapeutic nanoconjugate and determination of Drug/Nanoparticle Ratio

The products obtained after the T22-GFP-H6-FdU synthesis reaction were characterized using mass spectroscopy to measure their molecular mass. The volume and size distribution of the nanoparticles was determined by dynamic light scattering at 633 nm (Zetasizer Nano ZS, Malvern Instruments Limited, Malvern, Worcestershire, UK). Nanoconjugate size was also measured using purified samples, diluted to 0.2 mg/mL and contrasted by evaporation of 1 nm platinum layer in carbon-coated grids, before being visualized in a Hitachi H-7000 transmission electron microscope (TEM), as described (Unzueta 2012b). The drug to nanoparticle ratio was obtained by analyzing the UV spectra of T22-GFP-H6 and T22-GFP-H6-FdU nanoconjugate and calculating the number of FdU molecules per T22-GFP-H6 nanoparticle.

T22-GFP-H6-FdU internalization, CXCR4 specificity and cytotoxicity in CXCR4$^+$ HeLa cells

We assessed T22-GFP-H6-FdU capacity to internalize in a second cell type, the CXCR4$^+$ HeLa human cervical carcinoma cell line (ATCC® CCL-2™, Manassas, VA, USA), cultured in Minimum Essential Medium medium supplemented with 10% FBS and 2 mM Glutamax (Gibco, Waltham, MA, USA), by exposing cells for 1 hour to 1µM T22-GFP-H6-FdU concentration and measuring the green fluorescence emitted by the internalized cells in the flow cytometer FACS-Canto system (Becton Dickinson, Franklin Lakes, NJ, USA). To assess specificity for CXCR4 receptor-mediated internalization, we performed competition studies incubating CXCR4$^+$ HeLa cells with the CXCR4 antagonist AMD3100 (octahydrochloride hydrate, Sigma-Aldrich) in a 1:10 (Protein:antagonist) molar ratio for 1h before exposure to the nanoconjugate, as previously described for the T22-GFP-H6.

We assessed T22-GFP-H6-FdU subcellular localization using confocal microscopy. Briefly, cells were grown on culture dishes (Mat-Tek Co., Ashland, MA, USA); then, T22-GFP-H6-FdU was added in OptiPro medium supplemented with L-Glutamine. Nuclei were labeled with Hoechst 33342 (Life Technologies, Carlsbad, CA, USA) and plasma membranes with CellMaskTM Deep Red (Life Technologies) as described (Unzueta et al, 2012b). Micrographs were taken by TCS-SP5 confocal laser scanning microscopy (Leica Microsystems Wetzlar,Germany) and 3D models of T22-GFP-H6-FdU localization were generated using Imaris software (Bitplane, Zurich, Switzerland).

We also studied T22-GFP-H6-FdU cytotoxic activity using the MTT metabolic test (Roche, Basel, Switzerland). To that purpose, we exposed CXCR4$^+$ HeLa cells to T22-GFP-H6-FdU at 1-1,000nM concentration range and measured their viability at 48 hours as compared to equimolecular concentrations or free oligo-FdU. Afterwards, we constructed a graphic displaying the linearized T22-GFP-H6-FdU dose-response trend line representation to compare cell viability for both compounds. Reduction of cell viability was also determined by optical microscope images of HeLa cells exposed to 1µM T22-GFP-H6-FdU for 48 h, as compared to T22-GFP-H6 or free FdU.

T22-GFP-H6-FdU induction of DSBs and proteolyzed PARP in the subcutaneous CXCR4$^+$ SW1417 tumor model

We used the SC CXCR4$^+$ SW1417 CRC model in NOD/SCID mice to assess induction of double strand breaks (DSBs) and activation of PARP to evaluate induction of single-strand breaks (SSBs) shortly (at 30min, 1h and 5h) after the administration of a single 100µg dose of T22-GFP-H6-FdU as an i.v. bolus. We compare the nanoconjugate-indcued genotoxic damage with that observed in free oligo-FdU-treated (unconjugated free drug) after treatment with an equimolar dose or Buffer-treated tumors. At necropsy, we resected the tumors and performed immunohistochemistry (IHC) staining using either an anti-γH2AX mAb (1:400, Novus Biologicals, Cambridge, UK) or anti-proteolyzed PARP (anti-PARP p85 fragment; 1:200, Promega, Madison, WI, USA). We counted the number of cells stained cells, for either γH2AX or proteolyzed PARP, per high power field (400x magnification) in 5 different tumor sections from each mouse (N=25; 5 mice/group; 5 fields/mouse)

Evaluation of the induction of regression of established metastasis in an orthotopic CXCR4$^+$ SW1417 cancer model

We used the CXCR4$^+$ SW1417 orthotopic CCR model in *Swiss nude* mice to evaluate the possible inhibition of established metastasis. We started T22-GFP-H6-FdU administration two month after tumor cell implantation (when metastases were already present in mice, as measured by bioluminescence emission using the IVIS spectrum). To this purpose, we randomized 40 *Swiss nude* mice into four groups (buffer, T22-GFP-H6-FdU, T22-GFP-H6 and free FdU pentamer (n=10/each group). We administered them an i.v. dose of the corresponding compound (T22-GFP-H6-FdU: 20ug, T22-GFP-H6: 20ug, free oligo-FdU at equimolecular doses or Buffer) every three days for a total of 10 doses. The experiment was finished when the first animal of the Buffer-treated group was euthanized because of intestinal obstruction induced by primary tumor growth.

Evaluation of metastasis prevention in the orthotopic CXCR4$^+$ SW1417 cancer model and determination of the CXCR4$^+$ cancer cell fraction in cancer tissues at the end of treatment

We generated an efficient metastatic model, in *NOD/SCID* mice, that received an intracecal microinjection (ORT) of SW1417-luc CRC cells disaggregated from SC tumours previously generated in a different cohort of *NOD/SCID* mice. Briefly, when SC tumors reached a volume of 700 mm^3, mice were sacrificed by cervical dislocation and tumours were excised, discarding the necrotic areas, and three hundred mg of viable tumour tissue was then cut into pieces and disaggregated in a mix of 0.05% trypsin (Invitrogen) and 100 mg/ml DNase (Sigma-Aldrich). The mix was pipetted 30 times, using a 10 ml pipette, and incubated for 10 minutes at 37°C with shaking. It was then re-pipetted 30 times, using 10 ml, 3 ml and 1 ml pipettes, and re-incubated for 5 minutes at 37°C with shaking. The obtained SW1417 single-cell suspensions were filtered through a cell strainer and centrifuged at 1,000g for 10 minutes before counting the cells. We then microinjected 2×10^6 cells, previously grown in culture and resuspended in 50 µl of media, in the cecum of each mouse.

We used the SC+ORT metastatic SW1417 CRC model developed in *NOD/SCID* to evaluate the capacity of the nanoconjugate for metastasis prevention. We ramdomized mice into three groups: buffer (n=11), T22-GFP-H6-FdU (n=12) and free oligo-FdU (n=11) and administered repeated i.v. boluses at equimolecular doses, as follows: T22-GFP-H6-FdU 20ug, free oligo-FdU: 2.6 nmols, or buffer), every three days (q3d) for a total of 12 doses. We initiated the T22-GFP-H6-FdU administration one week after tumor cell implantation before metastatic dissemination had occurred (Appendix Fig S5). The experiment was finished when the first animal of the Buffer-treated group required to be euthanized. At necropsy, we recorded the number and size of visible metastases in all organs per mouse, counted *ex vivo* the number of metastatic foci that emitted bioluminiscence in the target organs, using the IVIS® 200-Spectrum, and performed the histopathological and immunohistochemical analyses to confirm location and number of metastases. We also evaluated CXCR4 expression by IHC, using the described anti-CXCR4 antibody, in primary tumor and metastatic foci at the different organs affected by metastases (peritoneum, liver, lung and lymph nodes). We quantified the fraction of CXCR4$^+$ cancer cells remaining in tumor tissue (CXCR4$^+$ CCF) after

treatment. The obtained results were used to study a possible correlation between $CXCR4^+$ CCF and antimetastatic effect at the different sites.

T22-GFP-H6-FdU biodistribution and toxicity in bone marrow and circulating blood cells

We used mice bearing subcutaneous $CXCR4^+$ SW1417 CRC tumors to assess T22-GFP-H6-FdU uptake (measuring green fluorescence emission) in bone marrow and circulating blood cells 5h after the administration of T22-GFP-H6-FdU at a range of 10-100 µg doses. At necropsy, bone marrow was extracted and registered *ex vivo* in the IVIS® 200-Spectrum equipment. Mouse blood was collected by punction in the maxillary plexus. The erythrocytes, leucocytes and platelets were isolated by the Ficoll density gradient method using the standard protocol. Further, pellets were obtained by centrifugation of the isolated cell extracts at 600g, 10min and then recorded to measure fluorescence using IVIS® 200-Spectrum equipment. Toxicity was evaluated in H&E stained bone marrow slides.

Statistical analysis

Sample size was defined on the basis of previous preliminary experiments. No animals nor samples were excluded from the analyses. Randomization of animals into control and experimental groups were performed using the SPSS program. Histology and immunohistochemical samples were coded so that the researcher that analysed them did not know to which group they belong to. Normal distribution of the data was tested using the Shapiro-wilk test. The homeogeneity of the variance between groups was tested using the Levene´s test. We used the Fisher's exact test to analyze possible differences between control and experimental groups of affected mice regarding metastatic rates at the different organs. The non-parametric tests, Kruskal-Wallis and post-hoc pairwise Mann-Whitney U two-sided tests were used to compare number and size of metastatic foci in the affected organs among groups. All quantitative values were expressed as mean±s.e.m. All statistical tests were performed using SPSS version 11.0 (IBM, New York, USA). Differences among groups was considered significant at a $P < 0.05$.

Appendix Table S1. Number of mice with undetectable metastases (Mets-free) and number of mice bearing metastatic foci (Mets +) at the end of the prevention of metastasis experiment in the T22-GFP-H6-FdU, free oligo-FdU or Buffer-treated groups, when using both, the sw1417 or M5 colorectal metastatic models.

**SW1417 model**	Primary tumor *	**Metastasis ****									
		Mets positive or mets-free mice / total number of mice (%)								Total # of mice with or without LVm, LGm and PTNm	
**Group**	**Tumor +** mice / / Total # Mice (%)	**Lymph nodes mets (LNm)**		**Liver mets (LVm)**		**Lung mets (LGm)**		**Peritoneal mets (PTNm)**			
		Mets free mice/total (%)	Mets+ mice/total (%)	Mets free mice/total (%)	Mets+ mice/total (%)	Mets free mice/total (%)	Mets+ mice/total (%)	Mets free mice/total (%)	Mets+ mice/total (%)	Total # of Mets free mice/group	Total # of Mets+ mice/group
**Buffer**	11/11 (100)	0/11 (0)	11/11 (100)	7/11 (64)	4/11 (36)	3/11 (27)	8/11 (73)[b]	4/11[64]	7/11[64][d]	14	19[e]
**Free oligo-FdU**	11/11 (100)	0/11 (0)	11/11 (100)	5/11 (45)	6/11 (55)[a]	5/11 (45)	6/11 (55)[c]	6/11[45]	5/11[45]	16	17[f]
**T22-GFP-H6-FdU**	12/12 (100)	3/12 (25)	9/12 (75)	10/12 (83)	2/12 (17)[a]	10/12(83)	2/12 (17)[b,c]	10/12 [17]	2/12 [17][d]	30	6[e,f]

**M5 patient-derived model**	Primary tumor *	**Metastasis ****									
		Mets positive or mets-free mice / total number of mice (%)								Total # of mice with or without LVm, LGm and PTNm	
**Group**	**Tumor +** mice / / Total # Mice (%)	**Lymph nodes mets (LNm)**		**Liver mets (LVm)**		**Lung mets (LGm)**		**Peritoneal mets (PTNm)**			
		Mets free mice/total (%)	Mets+ mice/total (%)	Mets free mice/total (%)	Mets+ mice/total (%)	Mets free mice/total (%)	Mets+ mice/total (%)	Mets free mice/total (%)	Mets+ mice/total (%)	Total # of Mets free mice/group	Total # of Mets+ mice/group
**Buffer**	6/6 (100)	0/6 (0)	6/6 (100)	0/6 (0)	6/6 (100)[g]	0/6 (0)	6/6 (100)	0/6 (0)	6/6 (100)	0	18[h]
**Free oligo-FdU**	7/7 (100)	0/7 (0)	7/7 (100)	1/7 (15)	6/7 (85)	2/7 (30)	5/7 (70)	1/7 (15)	6/7 (85)	4	17[i]
**T22-GFP-H6-FdU**	8/8 (100)	0/8 (0)	8/8 (100)	5/8 (63)	3/8 (37)[g]	4/8 (50)	4/8 (50)	3/8 (37)	5/8 (62)	12	12[h,i]

∞ * number of mice bearing a primary tumor out of the total number of mice per group
∞ ** number of mice affected by metastases out of the total number of mice per group
[a]p=0.05; [b]p=0.012; [c]p=0.05; [d]p=0.036; [e]p=0.0005; [f]p=0.004
[g]p=0.031; [h]p=0.0003; [i]p=0.05;

Appendix Fig. S1

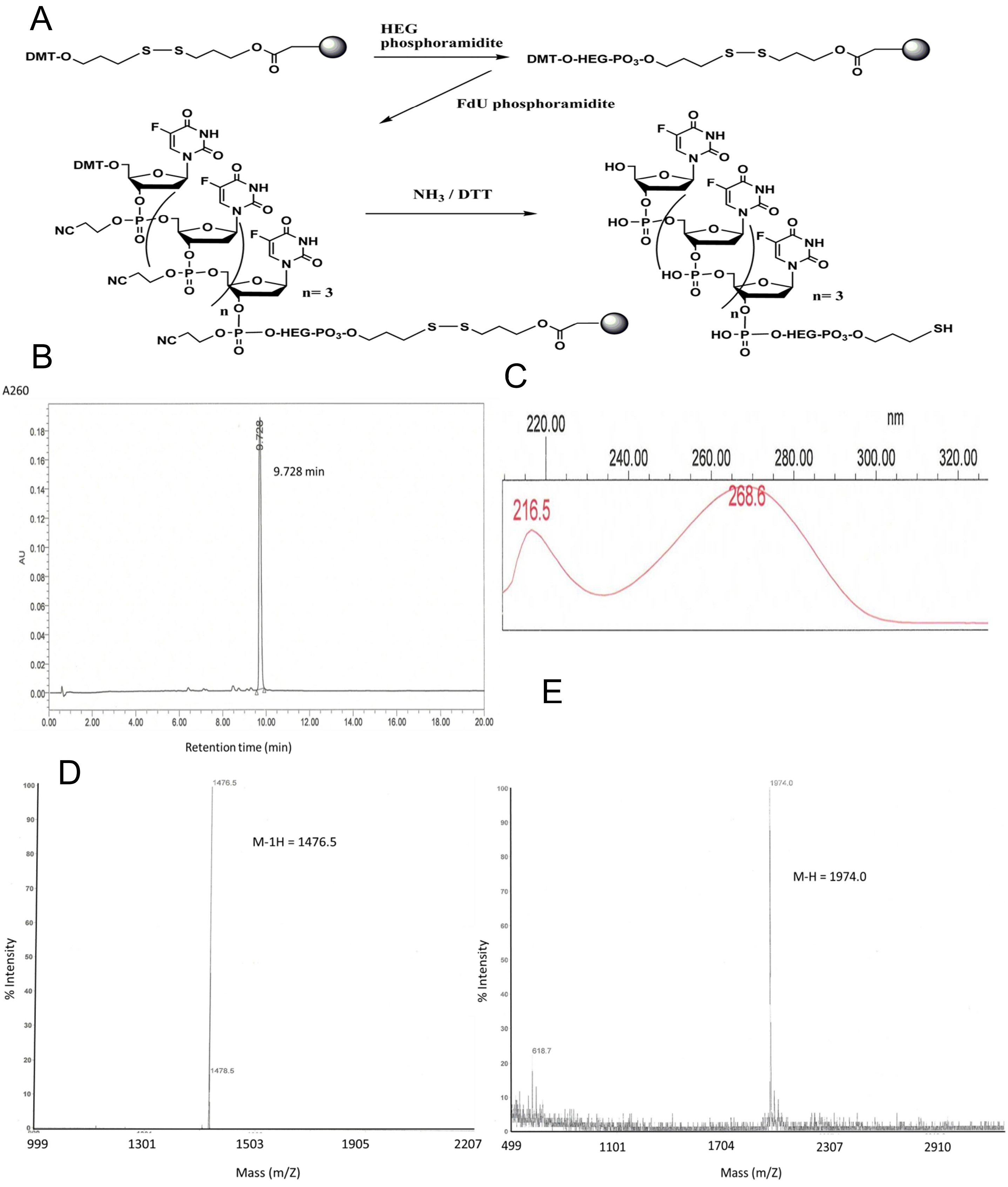

Appendix Figure S1. Synthesis of thiol functionalized oligo-FdU and physicochemical characterization

(**A**) Synthesis of Oligo-FdU functionalized with thiol: the pentamer oligo-$(FdU)_5$-SH (oligo-FdU) was synthesized in 1 µmol scale on an RNA/DNA synthesizer using β-cyanoethylphosphoramidite chemistry. 3'-Thiol-modifier C3 controlled pore glass (CPG) (Link Technologies) was used as solid support for the synthesis. First the hexaethyleneglycol (HEG) phosphoramidite (Glen Research) was incorporated. Then, the synthesis was completed by repetitive additions of the dimethoxytrityl (DMT)-protected-5-fluoro-2'-deoxyuridinephosphoramidite unit. After assembling of the sequence, oligonucleotide support was treated with aqueous ammonia (32%) with 0.1 M DTT (1,4- dithiothreitol) for 2h at room temperature. The ammonia solutions were concentrated to dryness and the product was desalted on NAP-10 (Sephadex G-25) columns eluted with water prior to use. Free oligo-FdU synthesis: Control pentamer oligo-FdU without HEG and thiol groups was prepared as before but using 3'-succinyl-FdU controlled pore glass as solid support. Finally, the oligonucleotide was deprotected with aqueous ammonia (32%) for 2h at room temperature.

(**B**) Physicochemical characterization of the $(FdU)_5$-HEG-SH. HPLC analysis of pentamer FdU-HEG-SH (Conditions: X-bridgeTM OST C18 (10x50 mm, 2.5 µm); 20 min linear gradient from 0% to 40%, flow rate 2 mL/min; solution A was 5% ACN in 0.1 M aqueous triethylammonium acetate(TEAA) and B 70% ACN in 0.1 M aqueous TEAA.

(**C**) UV spectra of pentamer $(FdU)_5$-HEG-SH. The pentamer was quantified by absorption at 260 nm.

(**D**) MS spectrum (MALDI-TOF) of control pentamer FdU (free oligo-FdU). M calculated 1478.1 M found 1476.5.

(**E**) MS spectrum (MALDI-TOF) of pentamer $(FdU)_5$-HEG-SH (oligo-FdU). M calculated 1976.2 M found 1974.0.

Appendix Fig. S2

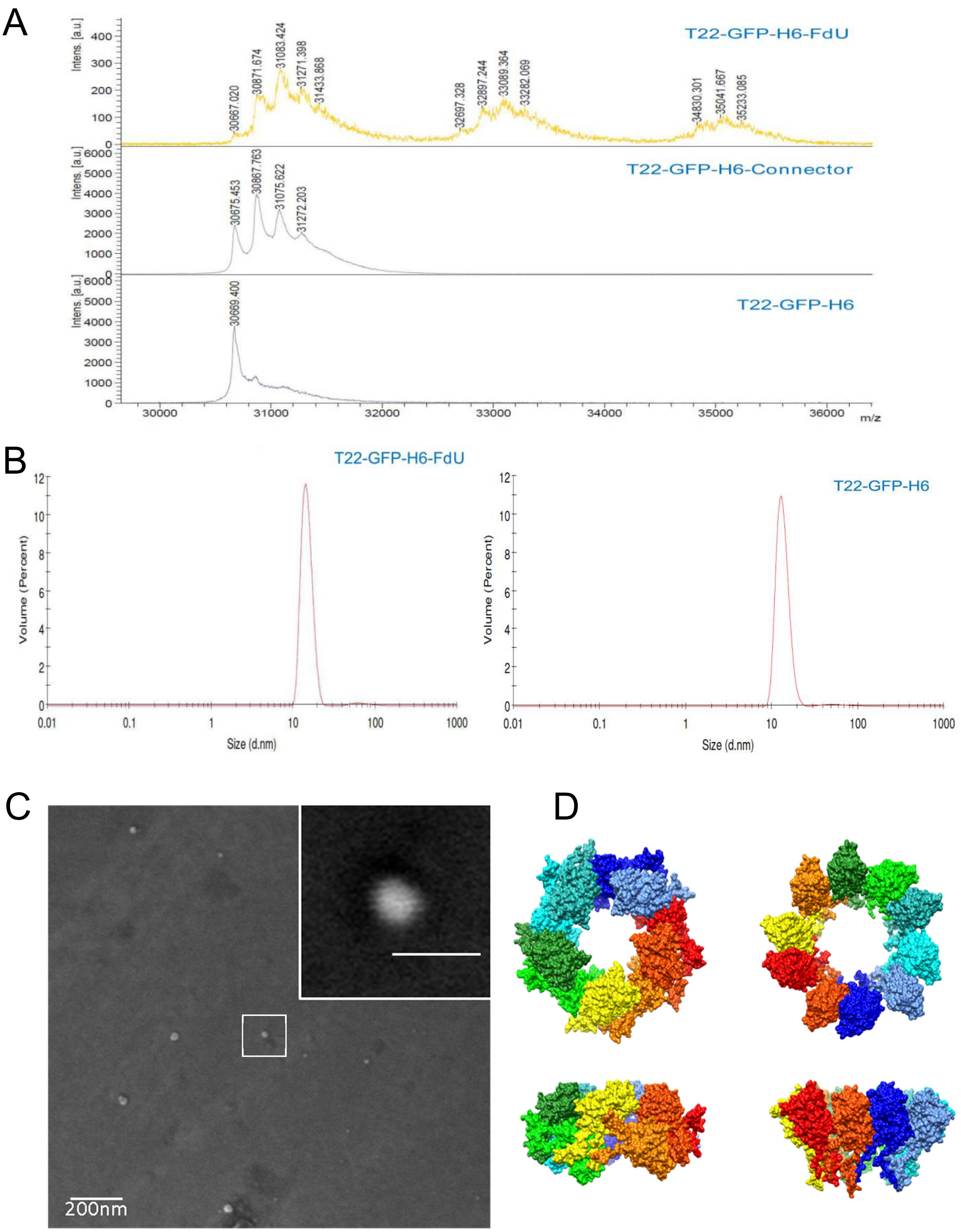

Appendix Figure S2. Physicochemical characterization of the T22-GFP-H6-FdU nanoconjugate and drug to nanoparticle ratio

(**A**) The analysis of the products of the conjugation was performed by MALDI-TOF spectra. Mass spectrometry of the T22-GFP-H6-FdU conjugation products identifying the molecular mass of the products carrying 1 oligo-FdU or 2 oligo-FdU payloads, the unconjugated T22-GFP-H6 protein and the linker-conjugated T22-GFP-H6.

(**B**) T22-GFP-H6-FdU size as determined by dynamic light scattering, as compared to T22-GFP-H6 nanoparticle.

(**C**) Representative T22-GFP-H6-FdU image as detected by transmission electron microscopy.

(**D**) Molecular modelling of T22-GFP-H6-FdU self-assembled nanoparticle (source: Rueda et al, (2015)). Printed with permission from John Wiley & Sons).

Appendix Fig. S3

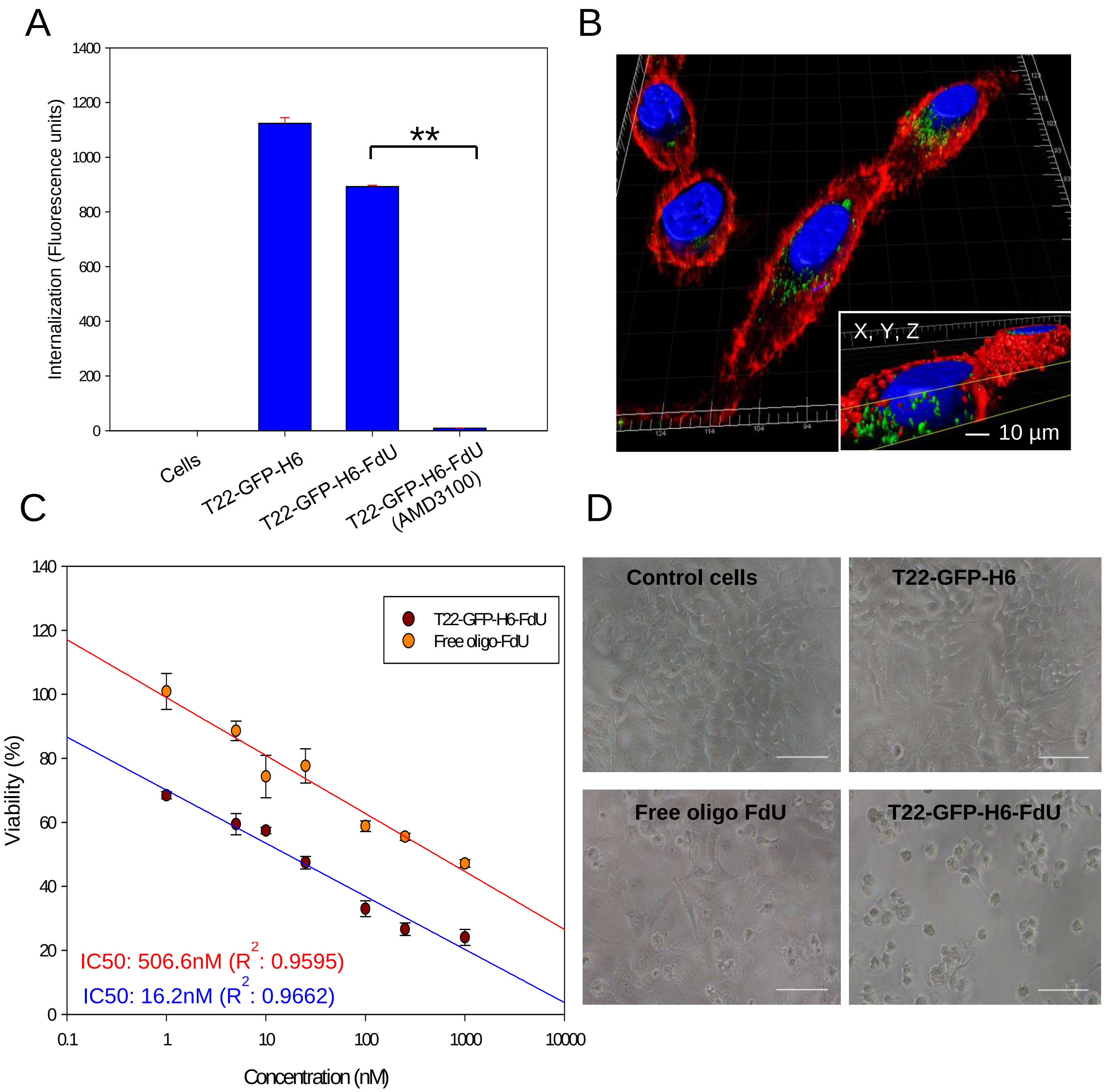

Appendix Figure S3. T22-GFP-H6-FdU selectively internalizes and kills $CXCR4^{+}$ HeLa cells in vitro

(**A**) T22-GFP-H6-FdU nanoconjugate or T22-GFP-H6 internalization in HeLa cells detected as emitted fluorescence in by flow cytometry after 1 hour cell exposure at 1 µM concentration. Complete internalization block obtained by pre-treatment of cells with the CXCR4 antagonist AMD3100.

(**B**) Intracellular trafficking of T22-GFP-H6-FdU in HeLa cells by confocal microscopy after exposure at 1 µM for 24 h. Data expressed as mean±s.e.m of 3 independent experiments performed in duplicate. Significant difference between T22-GFP-H6-FdU and T22-GFP-H6-FdU + AMD3100 at p= 0.000. The green staining corresponds to GFP containing nanoconjugates and the red staining corresponds to plasma cell membranes stained with a red dye (CellMask™), whereas cell nucleus was stained in blue with hoescht dye. The inset shows details of the intracellular localization of nanostructured, fluorescent entities, in an isosurface representation within a three-dimensional volumetric x-y-z data field

(**C**) Linearized T22-GFP-H6-FdU dose-response trend line representation compared with free oligo-FdU exposure. Antitumor effect was measured as HeLa cell viability by MTT after 72 h exposure as the described concentrations.

(**D**) Reduction of cell viability as recorded by optical microscope images of HeLa cells exposed to T22-GFP-H6-FdU nanconjugates for 48 h, as compared to T22-GFP-H6 or free oligo-FdU. Scale bar, 100 µm.

Appendix Fig. S4

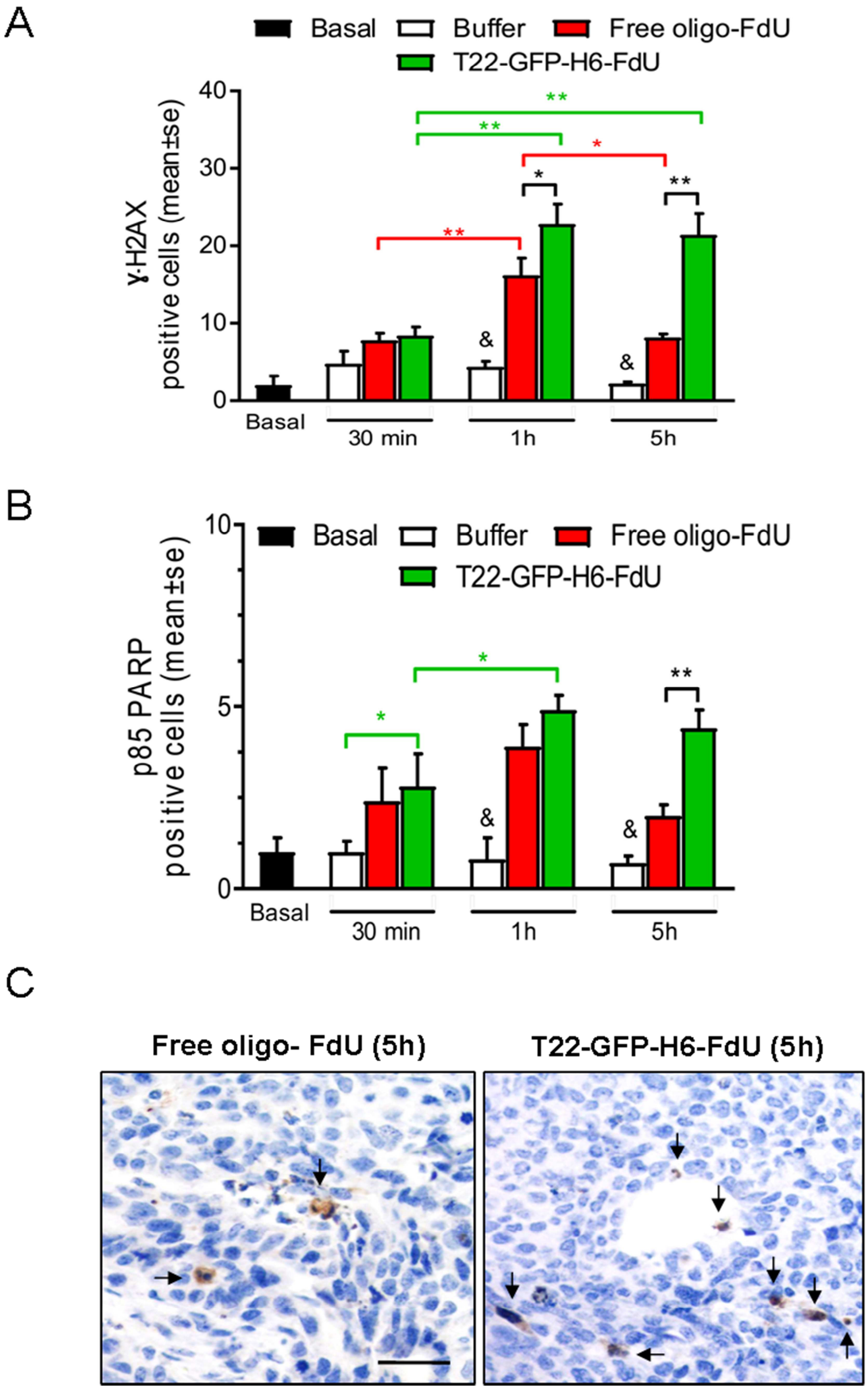

Appendix Figure S4. Induction of genotoxic damage in SW1417 subcutaneous tumors at early time points after T22-GFP-H6-FdU treatment

(A, B) Quantitation of the number of cells IHC stained with anti-γH2AX to assess double-strand-break (DSBs) induction (A) or the number of cells IHC stained with anti-PARP to assess PARP proteolysis and single-strand-break (SSBs) induction in SW1417 subcutaneous tumors at 30min, 1h and 5h after the treatment of mice with a single iv 100ug dose of T22-GFP-H6-FdU, and its comparison to the DNA damage induced with equimolar doses of free oligo-FdU or Buffer-treated mice. (N=25; 5 mice/group; 5 tumor fields/mouse). Scale bar, 50 µm. Data expressed as mean±s.e.m.

Parameter comparison at different time points (30 min, 1h, 5h) **between groups:** (**B**: Buffer; **F**: Free oligo-FdU; **T-F**: T22-GFP-H6-FdU).

-**ɣ-H2AX quantification** between groups at specific time points: **P values for Statistical Differences:** B1h vs F1h, P= 0.009; B5h vs F5h, P= 0.007; B1h vs T-F1h, P= 0.009; B5h vs T-F5h, P=0.006; F1h vs T-F1h, P= 0.03; F5h vs T-F5h, P= 0.001

-**ɣ-H2AX quantification** between time points within one group: **P values for Statistical Differences:** -T-F30min vs T-F1h, P= 0.03; T-F30min vs T-F5h, P= 0.04;

-**P85 PARP quantification** between groups at specific time points: **P values for Statistical Differences:** B1h vs F1h, P= 0.000; B5h vs F5h, P= 0.006; B30min vs T-F30min, P= 0.008; B1h vs T-F1h, P= 0.000; B5h vs T-F5h P=0.000; T-F5h vs F5h, P= 0.001

-**P85 PARP quantification** between time points within the same group: **P values for Statistical Differences:** T-F30min vs T-F1h, P= 0.03;

(C) Representative IHC-stained microphotographs of tumor cells stained with anti-PARP 5h after treatment with T22-GFP-H6-FdU, free oligo-FdU or Buffer.

Note that representative microphotographs of tumor cells IHC-stained for anti-γH2AX 5h after treatment with T22-GFP-H6-FdU, free oligo-FdU or Buffer are depicted in Figure 3A of the main text.

Appendix Fig. S5

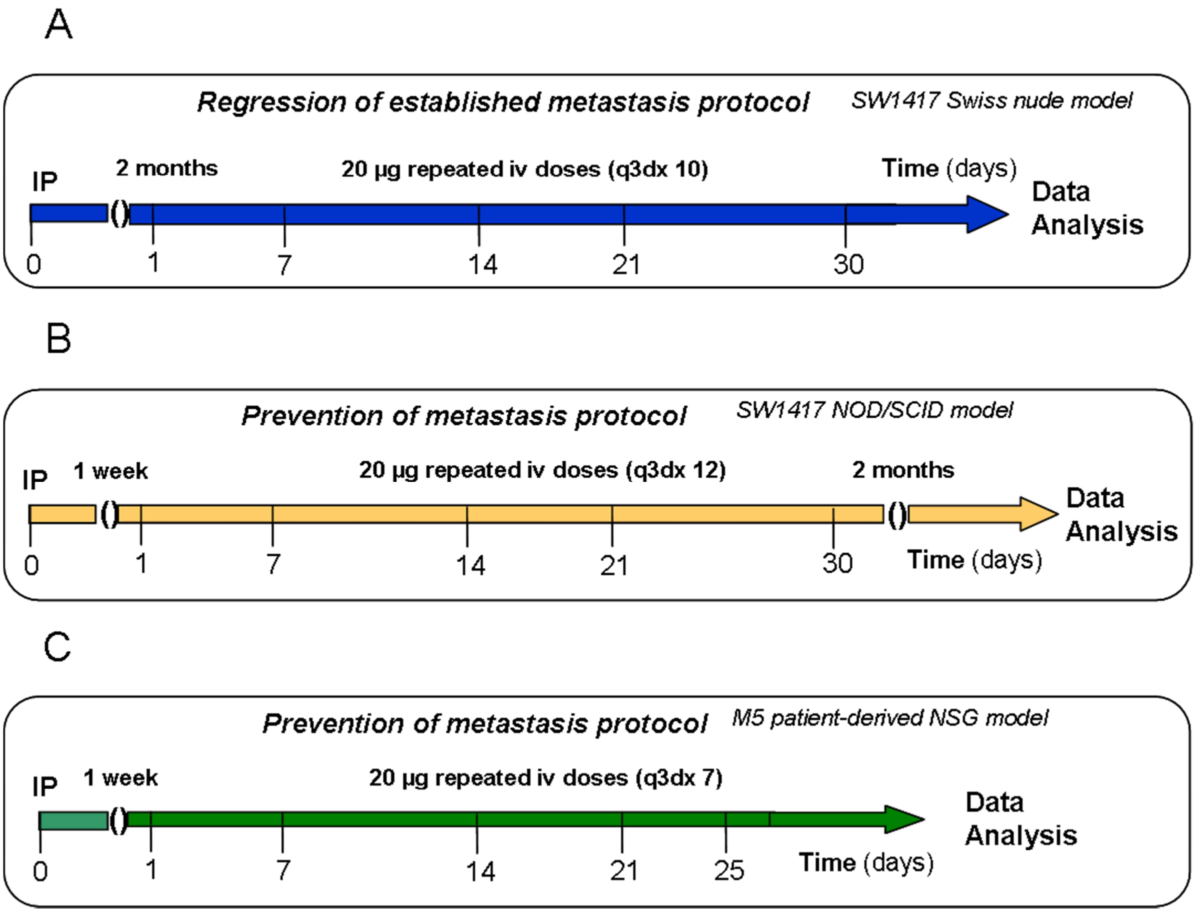

Appendix Figure S5. Mouse models, experimental setting and T22-GFP-H6-FdU dosage used in the regression and prevention of metastasis experiments

(**A**) Mouse model and protocol used in the regression of established metastasis study: Orthotopic $CXCR4^+$ SW1417 CRC model in Swiss nude mice. Experimental setting: T22-GFP-H6-FdU administration, at a dosage of 20µg q3d per 10 doses, was started 2 months after intracecal implantation of CRC cells (when established metastases are already present) to evaluate the nanoconjugate capacity to induce the regression of established metastases.

(**B**) Mouse model and protocol used in the prevention of metastasis study: Orthotopic $CXCR4^+$ SW1417 CRC model in NOD/SCID mice. Experimental setting: T22-GFP-H6-FdU administration, at a dosage of 20µg q3d per 12 doses, was started one week after intracecal implantation of CRC cells (when no metastases could yet develop), to evaluate the nanoconjugate capacity to prevent metastasis development.

(C) Mouse model and protocol used in the prevention of metastasis study: Orthotopic $CXCR4^+$ M5 patient-derived CRC model in NSG mice. Experimental setting: T22-GFP-H6-FdU was administered at a dosage of 20µg, q3d, per 7 doses, starting one week after cecal implantation of CRC cells.

In all three experiments, T22-GFP-H6-FdU antimetastatic effect is compared to that achieved by equimolecular doses of free oligo-FdU. Prior to initiating the prevention of metastasis experiments, we confirmed in separate mice that both, the cell line-derived and the patient-derived, mouse models lacked micro or macrometastases before treatment start. Prior to the initiation of the regression of metastasis experiment, we confirmed in separate mice that metastatic foci were already present before treatment start. Scale bar, 100 µm.

Appendix Fig. S6

A

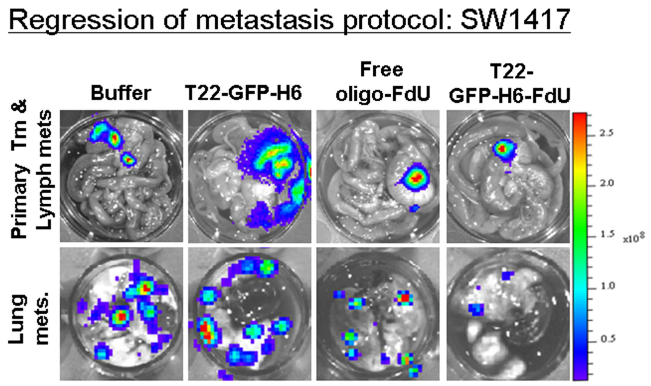

B

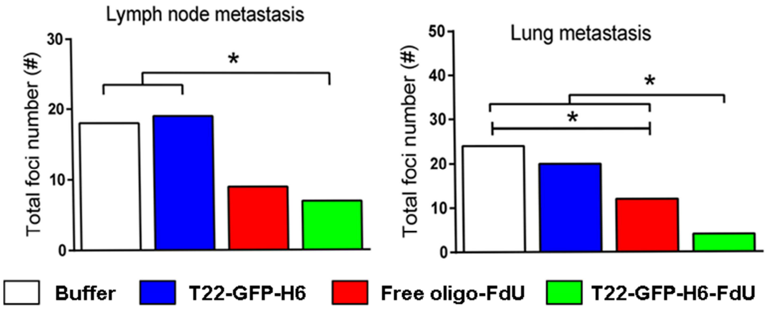

C

Regression of Metastasis Protocol

Groups	LN foci	Lung foci
**Buffer**	1.0 ± 0.3 ^{a}	4.0 ± 1.1 ^{c,d}
**T22-GFP-H6**	1.1 ± 0.2 ^{b}	1.7 ± 0.6 ^{e}
**Free oligo-FdU**	0.8 ± 0.2	2.0 ± 0.7 ^{c,f}
**T22-GFP-H6-FdU**	0.4 ± 0.1 ^{a,b}	0.7 ± 0.4 ^{d,e,f}

• Mean+SE foci number per mouse counted in 6 randomly chosen histology sections
•free-oligo-FdU: equimolecular doses of free oligo-FdU
•^{a}p=0.03; ^{b}p=0.01; ^{c}p=0.04; ^{d}p=0.03; ^{e}p=0.03, ^{f}p=0.04

D

Remaining CXCR4^{+} cancer cell Fraction (CXCR4^{+} CCF) in tumor tissue after T22-GFP-H6-FdU therapy

	Lymph mets	Lung mets
**Buffer**	12%	17%
**Free oligo-FdU**	10%	14%
**T22-GFP-H6-FdU**	13%	8%
Sensitivity to the nanoconjugate	*Resistant*	*Medium*

Appendix Figure S6. T22-GFP-H6-FdU induces the regression of established metastases in a CXCR4-dependent manner

(**A**) Repeated doses of T22-GFP-H6-FdU induced a higher inhibition of lung mets than an equimolecular dosage of free oligo-FdU, as measured by bioluminescence emission at the end of the regression of metastases experiment, whereas both compounds showed a similar level of inhibition of lymphatic mets in the $CXCR4^+$ SW1417 orthotopic model.

(**B**) T22-GFP-H6-FdU mice induced a higher reduction in the total number of lung mets than free oligo-FdU as recorded in H&E stained tissue sections at the end of the regression of metastasis experiment, whereas both compounds showed a similar level of inhibition of lymphatic mets. (N=60; 10 mice/group; 6 sections/mouse). See panel C for statistical analysis of the data.(**C**)T22-GFP-H6-FdU induced a significant reduction in the mean number of metastatic foci in lung, as compared to free oligo-FdU, as recorded in six histology sections per mouse. (N=60; 10 mice/group; 6 sections/mouse)

(**D**) T22-GFP-H6-FdU induces a high reduction in the $CXCR4^+$ cancer cell fraction ($CXCR4^+$ CCF) remaining at the end of treatment, as detected by anti-CXCR4 IHC, in lung metastatic tissue, whereas this reduction is not observed in lymph node mets tissue. Note that the reduction in $CXCR4^+$ CCF induced by T22-GFP-H6-FdU at the end of the experiment is only observed in lung mets, which show high sensitivity to the nanoconjugate.

Appendix Fig. S7

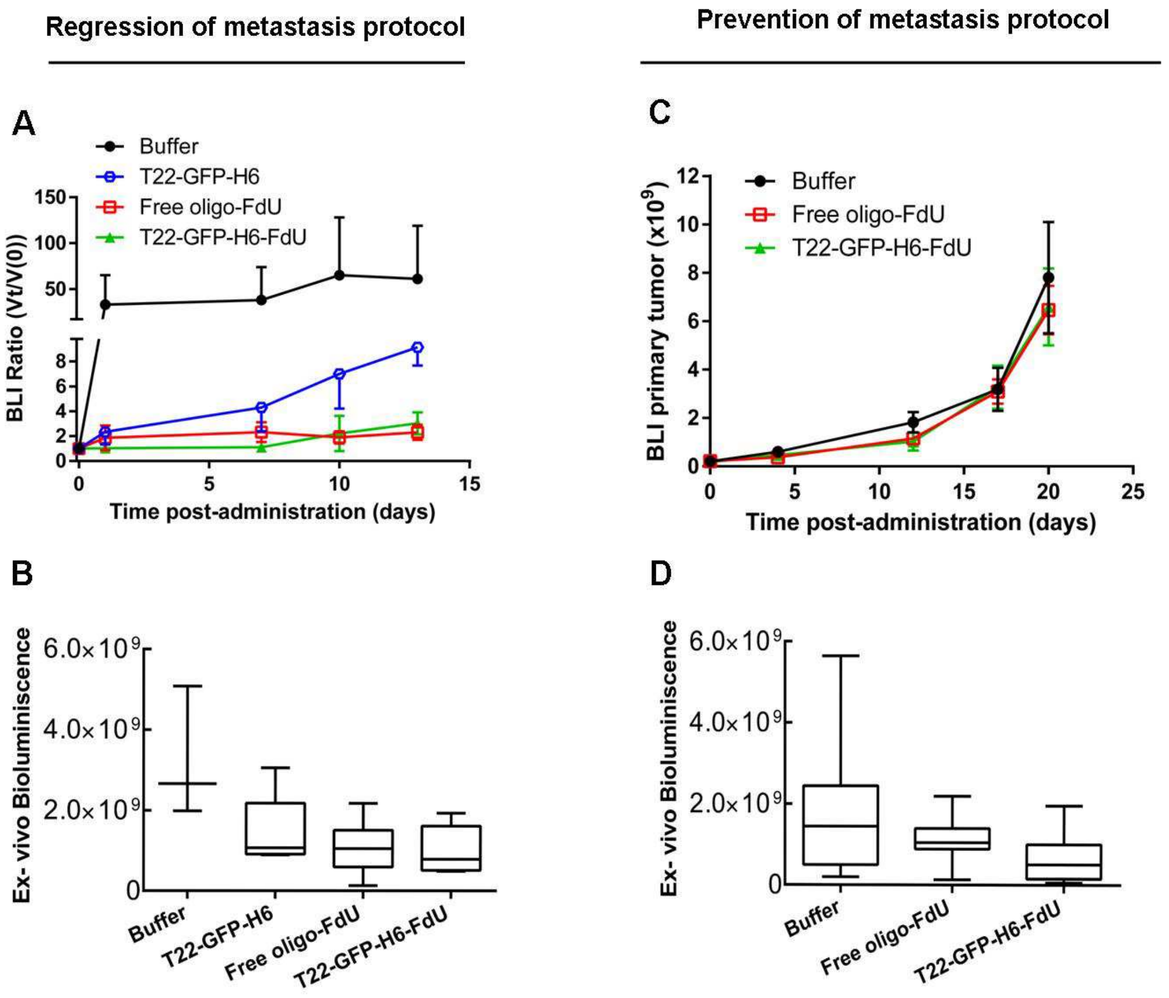

Appendix Figure S7. T22-GFP-H6-FdU inhibition of primary tumor growth

(**A**) In the SW1417 prevention of metastasis experiment, no significant inhibition in primary tumor growth was observed along the experimental time, as measured by in vivo bioluminescence emission, after T22-GFP-H6-FdU or free oligo-FdU treatment, as compared to buffer-treated mice. (N=10 mice/group)

(**B**) *Ex vivo* recording of bioluminescene emitted by the primary tumor after its resection at the end of the experiment showed also similar levels among T22-GFP-H6-FdU, free oligo-FdU and Buffer-treated mice. (N=10 mice/group)

(**C**) In the regression of established metastasis experiment, T22-GFP-H6-FdU inhibited primary tumor growth along the experimental time, as detected by *in vivo* bioluminescence emision (BLI), to levels similar to these achieved by free oligo-FdU. Both compounds significantly inhibited tumor growth as compared to buffer treated animals. (Buffer (N=11); Free oligo-FdU (N=12); T22-GFP-H6-FdU (N=12); see Table 1)

(**D**) *Ex vivo* recording of bioluminescence emitted by the primary tumor, after its resection at the end of the experiment, showed also similar levels between the T22-GFP-H6-FdU and free oligo-FdU mice (Buffer (N=11); Free oligo-FdU (N=12); T22-GFP-H6-FdU (N=12); see Table 1)Note that in contrast to the observed similar response in primary tumor after T22-GFP-H6-FdU or free-oligo-FdU treatment, the antimetastatic effect induced by T22-GFP-H6-FdU was significantly higher than that observed after free oligo-FdU treatment, especially regarding metastasis prevention (see Appendix Fig S8, Table 1 and Appendix Table S1).

Appendix Fig. S8

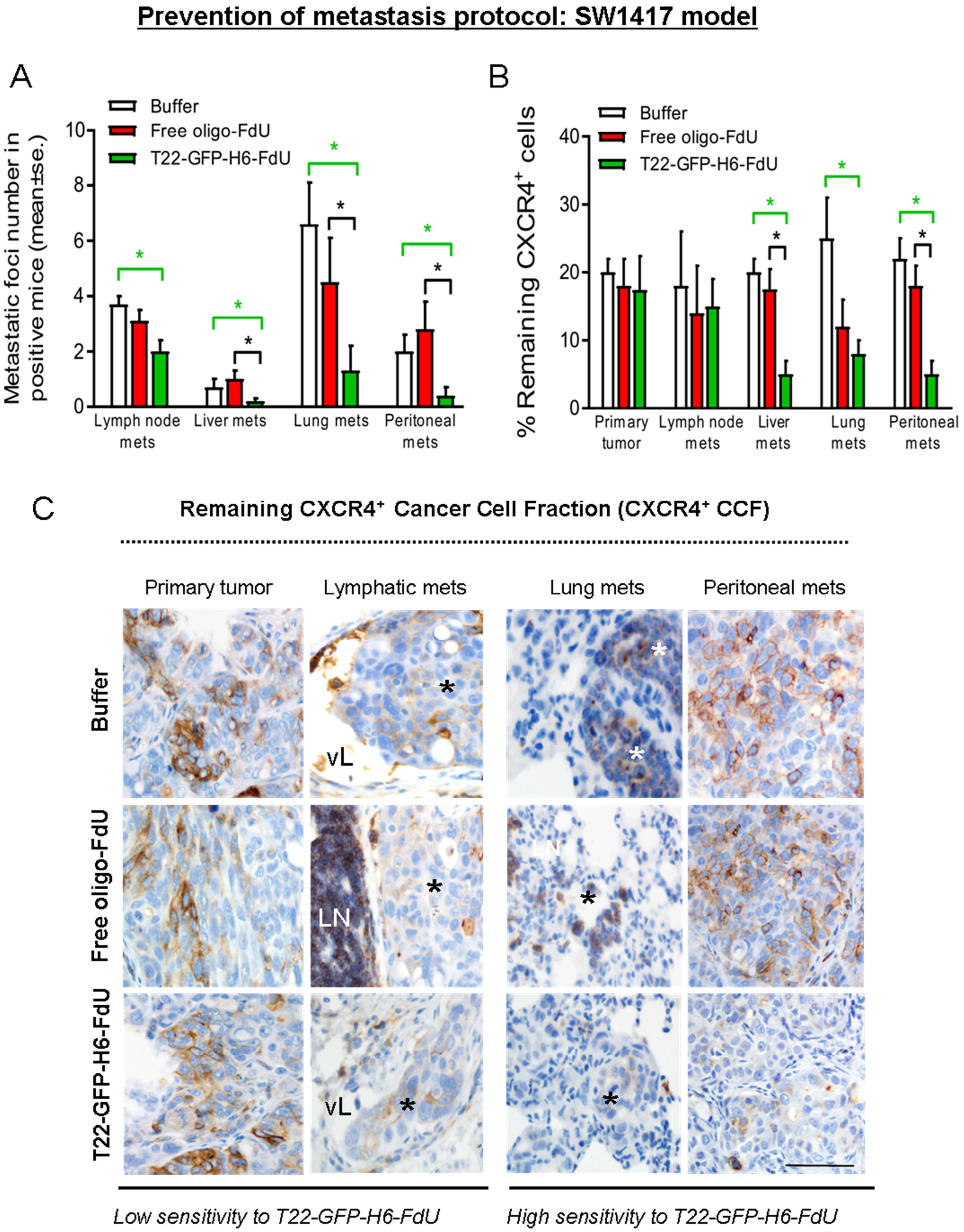

Appendix Figure S8. Reduction in CXCR4$^+$ cancer Cell Fraction induced by T22-GFP-H6-FdU in the SW1417 orthotopic model.

(**A**).T22-GFP-H6-FdU prevents metastases in the CXCR4$^+$ SW1417 orthotopic model by potently reducing the total and mean number of liver, lung and peritoneal mets, as recorded in H&E stained histology sections at the end of treatment, in comparison to free oligo-FdU. In contrast, the number of LN mets is not reduced after free oligo-FdU administration. See Table 1 for the recording of the percent of metastasis-free mice (mice with undetectable metastases at the end of treatment) with T22-GFP-H6-FdU and the induction of mean number foci and foci size in metastasis positive mice.

Metastatic sections analyzed per group: Buffer: N=33, 3 sections/mouse x 11 mice; **Free-oligo-FdU:** N=36, 3 sections/mouse x 12 mice; **T22-GFP-H6-FdU:** N=36, 3 sections/mouse x 12 mice. Data expressed as mean±s.e.m. See Table 1 for statistical analysis of the data.

(**B**) T22-GFP-H6-FdU induces a higher reduction of CXCR4$^+$ cancer cell fraction (CXCR4$^+$ CCF) in liver and peritoneal metastatic tissue, at the end of treatment, than free oligo-FdU, as measured by anti-CXCR4 IHC. In contrast, T22-GFP-H6-FdU or free oligo-FdU does not reduce the CXCR4$^+$ CCF in LN mets or primary tumor tissue after therapy.

Metastatic sections analyzed per group: Buffer: N=33, 3 sections/mouse x 11 mice; **Free-oligo-FdU:** N=36, 3 sections/mouse x 12 mice; **T22-GFP-H6-FdU:** N=36, 3 sections/mouse x 12 mice. Data expressed as mean±s.e.m. See Table 1 for statistical analysis of the data.

(**C**) Representative images of the reduction in CXCR4$^+$ CCF induced by T22-GFP-H6-FdU, as compared to free oligo-FdU at the end of therapy in the SW1417 cell line-derived CRC model. Note the correlation between the reduction in CXCR4$^+$ CCF induced by T22-GFP-H6-FdU and its antimetastatic effect (Table 1) at the different sites. The highest reduction in foci number and size occurs in peritoneal metastases, which shows the highest reduction in CXCR4$^+$ CCF. Scale bar, 100 μm. Asterisks: tumor tissue; vL: lymphatic vessel, LN: lymphatic metastasis.

Appendix Fig. S9

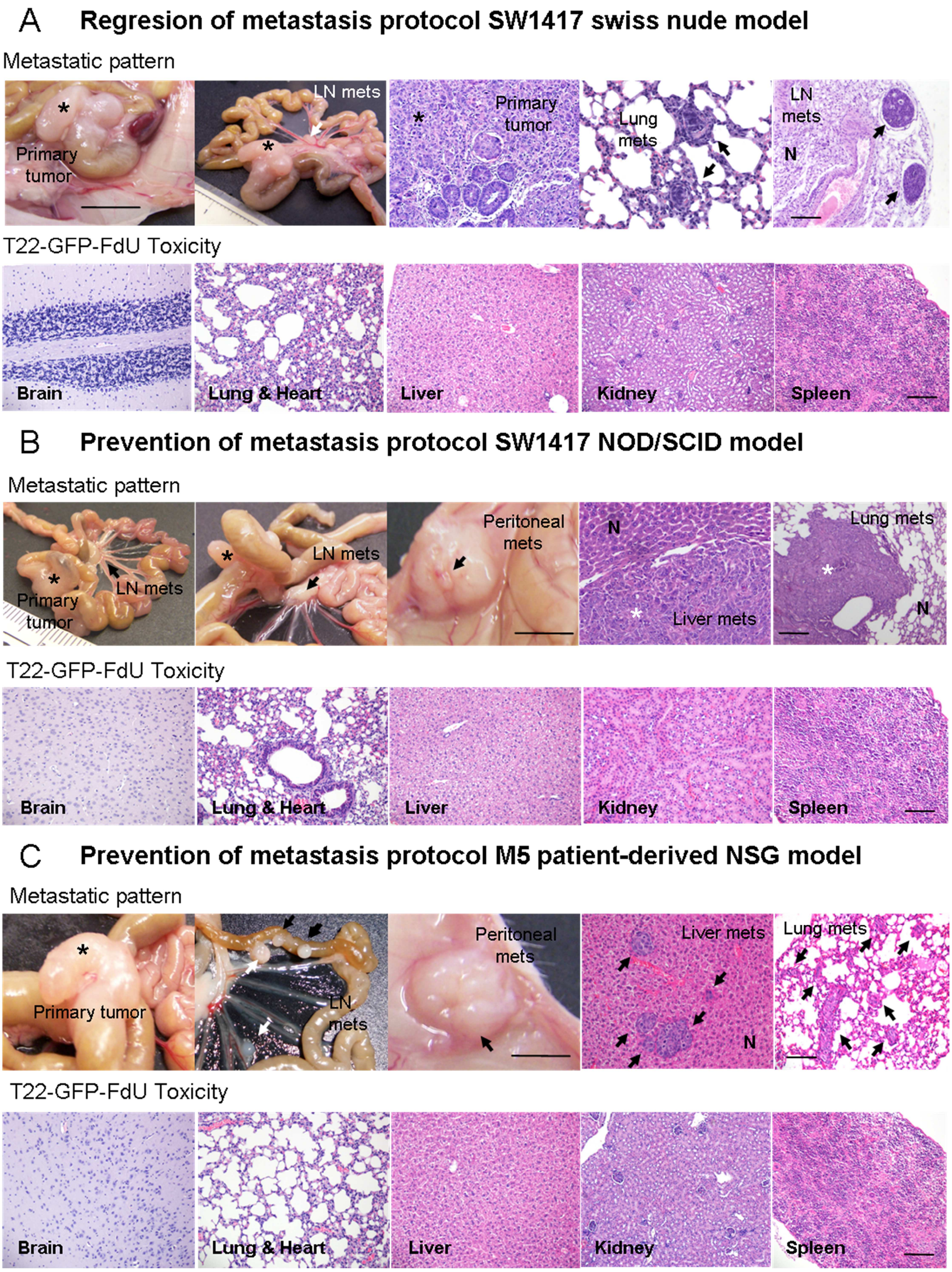

Appendix Figure S9. Metastatic pattern and toxicity of the orthotopic CCR mouse models used in the regression and prevention of metastasis experiments

(**A**) Mouse model used in the regression of established metastasis protocol: Direct orthotopic implantation of $CXCR4^+$ bioluminescent SW1417 CRC cells in *Swiss nude* mice generates metastases in the lymph nodes and lung. Note the lack of histological alterations observed in the described normal organs in this model at the end of the repeated T22-GFP-H6-FdU dose therapy administered. Scale bar, 1 cm.

(**B**) Mouse model used in the prevention of metastasis protocol: subcutaneous passage of $CXCR4^+$ bioluminescent SW1417 cells followed by orthotopic implantation of disaggregated tumor cells (SC+ORT) in *NOD/SCID* mice, which generates metastases in lymph node, liver, lung and peritoneum. Note the lack of histological alterations observed in the described normal organs in this model at the end of the repeated T22-GFP-H6-FdU dose therapy administered.

(**C**) Mouse model used in the prevention of metastasis protocol: subcutaneous tumors of the $CXCR4^+$ M5 patient-derived tumor-line was disaggregated. Isolated tumor cells were orthotopically implanted in *NSG* mice. This model is highly efficient in generating metastases in Lymph nodes, liver, lung and peritoneum. Note the lack of histological alterations observed in the described normal organs in this model at the end of the repeated dose regime of T22-GFP-H6-FdU administered.

Appendix Fig. S10

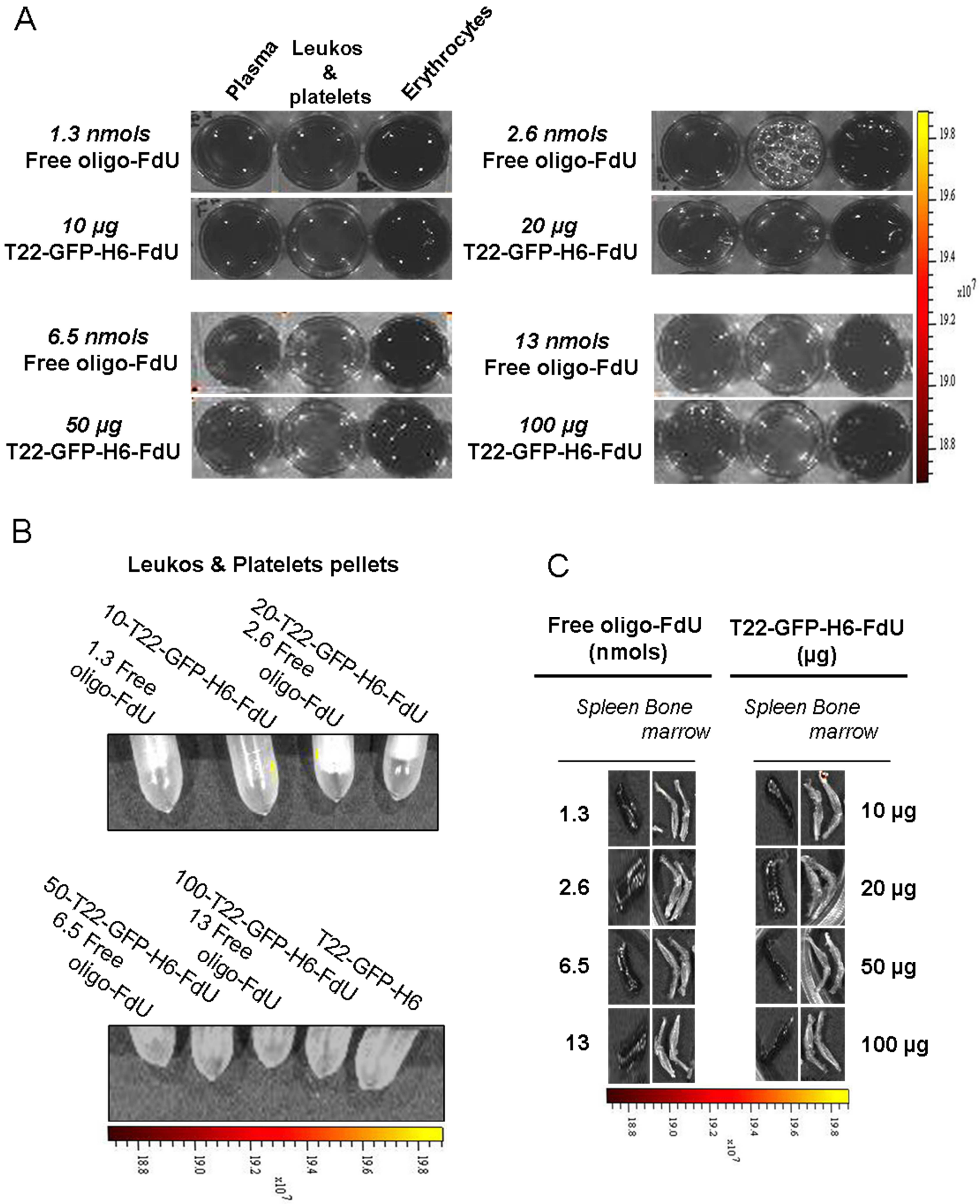

Appendix Figure S10. Undetectable T22-GFP-H6-FdU accumulation in bone marrow or circulating blood cells

(**A**) Lack of fluorescence emission in erythrocytes, leucocytes or platelets isolated from blood using a Ficoll density gradient 5h after the administration of T22-GFP-H6-FdU at doses in the 10-100 μg range, or control equimolecular doses of free FdU (1.3-13.0 nmol range). (N=5 mice/dose).

(**B**) Undetectable fluorescence emission observed in isolated leukocytes and platelet pellets after the Ficoll protocol. (N=5 mice/dose).

(**C**) Lack of fluorescence emission in spleen or bone marrow obtained 5h after the treatment of mice with single injections of T22-GFP-H6-FdU or control free oligo-FdU in the dose range described in (**A)**
